# Serum MMP-9 Diagnostics, Prognostics, and Activation in Acute Coronary Syndrome and Its Recurrence

**DOI:** 10.1007/s12265-018-9789-x

**Published:** 2018-01-18

**Authors:** Laura Lahdentausta, Jaakko Leskelä, Alina Winkelmann, Taina Tervahartiala, Timo Sorsa, Erkki Pesonen, Pirkko J. Pussinen

**Affiliations:** 1 Department of Oral and Maxillofacial Diseases, University of Helsinki and Helsinki University Hospital, Helsinki, Finland; 2 Department of Periodontology, Operative and Preventive Dentistry, University Hospital Bonn, Bonn, Germany; 3 Division of Periodontology, Department of Dental Medicine, Karolinska Institutet, Huddinge, Sweden; 4 Skåne University, Skåne, Sweden; 5 Department of Paediatrics, Division of Paediatric Cardiology, Skåne University Hospital, Lund, Sweden

**Keywords:** Atherosclerosis, Coronary artery disease, Serum biomarker, Cardiovascular diseases, Plaque rupture, Inflammation

## Abstract

Matrix metalloproteinase (MMP)-9 is crucial in atherosclerotic plaque rupture and tissue remodeling after a cardiac event. The balance between MMP-9 and endogenous inhibitor, tissue inhibitors of matrix metalloproteinase 1 (TIMP-1), is important in acute coronary syndrome (ACS). This is an age- and gender-matched case-control study of ACS (*N* = 669). Patients (45.7%) were resampled after recovery, and all were followed up for 6 years. The molecular forms of MMP-9 were investigated by gelatin zymography. Diagnostically, MMP-9 and the MMP-9/TIMP-1 molar ratio were associated with ACS (OR 5.81, 95% CI 2.65–12.76, and 4.96, 2.37–10.38). The MMP-9 concentrations decreased 49% during recovery (*p* < 0.001). The largest decrease of these biomarkers between acute and recovery phase (ΔMMP-9) protected the patients from major adverse cardiac events, especially the non-fatal events. The fatal events were associated with in vitro activatable MMP-9 levels (*p* = 0.028). Serum MMP-9 and the MMP-9/TIMP-1 molar ratio may be valuable in ACS diagnosis and prognosis. High serum MMP-9 activation potential is associated with poor cardiovascular outcome.

**Electronic supplementary material:**

The online version of this article (10.1007/s12265-018-9789-x) contains supplementary material, which is available to authorized users.

## Introduction

Atherosclerosis is a chronic inflammatory process of arteries [1, 2]. Matrix metalloproteinases (MMPs) destabilize atherosclerotic plaques by degrading extracellular matrix (ECM), especially in the shoulder regions. This may lead to plaque rupture and a fatal acute coronary syndrome (ACS) event. Inflammatory and oxidative mediators increase the amounts of MMPs [3]. MMPs enable leukocytes and inflammatory mediators to migrate across tissues [4], accelerating the development of pathogenic atherosclerotic plaques.

Matrix metalloproteinase-9 (MMP-9), also known as gelatinase B, is an enzyme that degrades mainly type IV collagen and elastin [5]. MMP-9 is secreted by various cell types, such as neutrophils, macrophages, endothelial cells, and smooth muscle cells. Interactions with specific tissue inhibitors of matrix metalloproteinases (TIMPs) determine the function of MMP-9 [6, 7] by binding to MMP at a molar equivalence [8]. Inactive, latent pro-form MMP-9 may be activated by reactive oxygen species (ROS), trypsin, chymotrypsin, or bacterial proteases [9]. In addition, MMP-9 can be activated chemically with APMA (*p*-aminophenylmercuric acetate) in vitro [10].

Significantly elevated plasma MMP-9 concentrations have been previously reported in ACS patients [11–13]. Elevated serum MMP-9 concentration was associated with plaque rupture when compared to stable angina pectoris patients [14]. However, there is no information available, if the MMP-9 concentrations decrease during the recovery or if the recovery levels associate with recurrent cardiovascular (CV) events. In general, only a few follow-up studies have been published. In one study, ST-elevation myocardial infarction (STEMI) patients showed higher serum MMP-9 and MMP-9/TIMP-1 values compared to subjects without coronary artery disease (CAD), but they had no prognostic value in a 2-year follow-up [15]. In another study with a longer follow-up (mean 4.1 years), elevated MMP-9 was associated with an adverse event [16].

In the present study, we determined serum MMP-9 and TIMP-1 concentrations and calculated their molecular ratio. The determinations were done on controls at baseline and on ACS patients in the acute and in the recovery phase. The patients were followed up for 6 years. Using these serum concentrations, our aims were to investigate (1) their association and diagnostic value in ACS in a cross sectional setting, (2) the difference between acute and recovery phase (i.e., Δ values), (3) their prognostic value by using the follow-up data, and (4) the relevance of MMP-9 activation degree in ACS.

## Materials and Methods

### Subjects and Diagnosis

Data was collected between March 1999 and April 2002 as described earlier [17–19]. Cases were ACS patients admitted in the heart intensive care unit at Lund University Hospital. The 343 patients included 108 unstable angina pectoris (UAP) and 235 acute myocardial infarction (AMI) patients. Forty-eight patients, who were invited to the study, chose not to participate and 21 patients died before the appointment. After a 6-month recovery period (median 350 days, IQR 434 days), 157 (45.8%) patients agreed to participate in the resampling.

The inclusion criteria for both cases and controls were age under 80 years, no cognitive intellectual disability, and no operations or chemotherapy within the previous 4 weeks. Twelve patients were excluded from the study because of aortic aneurysm, pulmonary embolism, myocarditis, pericarditis, unspecific precordial pain, or atrial fibrillation. Data on demographic factors were collected by a questionnaire. Patient history and use of medications were registered, and blood samples were drawn.

Cases were diagnosed with typical symptoms, laboratory measurements, and electrocardiogram (ECG). AMI and UAP were diagnosed according to prevailing criteria in 2000. AMI was diagnosed if the patient had two of the following criteria: chest pain related to exercise lasting over 20 min or changes in ECG, such as ST-elevations followed by T-wave inversion or new Q-waves, or an increase in Creatine kinase-MB (CK-MB) to more than twice the upper limit of the normal value (> 5 μg/l). UAP was diagnosed if two of following criteria were fulfilled: continuous chest pain, ST-segment depression in the ECG (<1 mm), or elevation of CK-MB (5 < CK-MB < 10 μg/l) or troponin T (0.05 < TnT < 0.10 μg/l).

The controls (*N* = 326) did not have previous coronary heart disease, stroke, or angina-like chest pain and were not taking medication for dyslipidemia, hypertension, or diabetes. They were selected from the same suburbs as the patients, and the groups were matched for gender and age ± 2 years. The control samples were collected and stored similarly as those of the cases.

All subjects of the study were followed up on average for 6 years (range 4.56–7.13) to the end of the study or to a major adverse cardiac event (MACE), i.e., cardiovascular death or hospitalization for an ACS event. During the follow-up, 150 patients suffered MACE, including 61 fatal and 89 non-fatal events. Among the patients, whose recovery samples were available, 63 patients suffered a MACE, including 14 fatal and 49 non-fatal events.

A subsample of patients who suffered a non-fatal (*N* = 23) or fatal (*N* = 7) MACE and whose both acute and recovery phase samples were available were randomly selected for gelatin zymography. Zymographies were run also on a set of controls (*N* = 28) that were matched for age and gender to the cases on a group level. The total number of samples in these analyses was 58. The patients in this subsample differed from the rest of the cases only as regards to systolic blood pressure (150 vs. 130 mmHg, *p* = 0.033).

### Laboratory Determinations

Serum without activators was collected from the patients within 24 h after the diagnosis. The research nurse collected 70 ml of blood from controls. The blood samples were transferred on ice to the central laboratory for centrifugation. All samples were frozen (− 20 °C) until laboratory analyses. In the in-house laboratory quality control, no significant effect of storage time on biomarker levels has been observed [20].

TIMP-1-ELISA (R&D Systems, Minneapolis, MN, USA) and MMP-9-ELISA (GE Healthcare UK Limited, Amersham Place, UK) were performed according to manufacturer’s instructions on diluted samples (1:10 in TIMP-1 and 1:20 in MMP-9). The inter-assay CV% of TIMP-1 was 8.2% (*N* = 12) and for MMP-9 9.2% (*N* = 12). The detection limits were 0.08 and 0.05 ng/ml, respectively. TIMP-1 can form a complex with MMP-9 in a 1:1 stoichiometry with a high affinity [21]. For calculation of MMP-9/TIMP-1, stoichiometric molecular ratio molecular weights of 92,000 g/mol (MMP-9) and 28,000 g/mol (TIMP-1) were used. The recovery phase levels of MMP-9 and MMP-9/TIMP-1 were subtracted from those of the acute phase to calculate the Δ levels.

Gelatin zymography was performed using a previously described technique [22] with minor modifications to explore activatable MMP-9 in vitro. The incubation time (16 h) and sample volume (1 μl) were based on pilot zymography analyses with self-cast gels using fluorescent gelatin substrate. Gelatin zymography analyses of selected samples were performed with commercial gels (BIO-RAD 10% Ready Gel® Zymogram Gel, 10 well, 50 μl, #161-1167, CA, USA). Experiments were conducted with serum samples as such and with samples processed with APMA for in vitro activation of MMP-9. Serum was incubated at a final concentration of 1 mM APMA at + 37 °C for 1 h. Subsequently, all samples were incubated in Laemmli buffer at room temperature for 1 h. After incubation, the samples were applied on gels and electrophoresis was performed in + 4 °C for 2 h. After electrophoresis, the gels were incubated at room temperature in the first wash buffer (50 mM Tris-HCl, pH 7.5, containing 2.5% Tween 80 and 0.02% NaN_3_) for 30 min, in the second buffer (50 mM Tris-HCl, pH 7.5, containing 2.5% Tween 80 and 0.02% NaN_3_ and supplemented with 1 μM ZnCl_2_, 5 mM CaCl_2_) at room temperature for further 30 min, and in the last buffer (the same as the second buffer but without 2.5% Tween 80) at 37 °C for 16 h. The gels were stained with 0.1% Coomassie Brilliant Blue and destained with 20% methanol/10% acetic acid. Low-range prestained SDS-PAGE standards (BIO-RAD, #161-0305, CA, USA) were used in every gel. MMP-9 (Proteaimmun 100 ng/μl) was used as a positive control in every gel, and the molecular weights of the gelatinolytic zones were compared to the positive control. The gels were scanned with LI-COR ODYSSEY (Lincoln, NE, USA), and data was analyzed with Image Studio software. The data was presented as arbitrary scanning units of intensities.

### Statistics

The distribution of variables was tested before statistical analysis. Normally distributed continuous variables are presented as means and standard deviations (SD). The statistical significance of the differences between the groups was tested by Student’s *t* test or ANOVA of logarithmically transformed values. Categorical variables were tested by chi-square test. The ELISA measurements of MMP-9 and TIMP-1 as well as the gelatin zymography results displayed a skewed distribution and are presented as medians and interquartile ranges (IQR). Statistical significance was tested by using non-parametric Mann-Whitney *U* test or Wilcoxon signed-rank test. The diagnostic sensitivity and specificity of MMP-9 and MMP-9/TIMP-1 were calculated by receiver operating characteristics (ROC) from logarithmically transformed values. Two different multivariate logistic regression models were used to determine the association of MMP-9 and MMP-9/TIMP-1 molar ratio with ACS. The first model was stratified for age and sex; the second model was stratified for age, sex, and adjusted for C-reactive protein (CRP), cholesterol, and smoking. For the follow-up data, we used Cox regression model adjusted for age and sex. MACE and its subgroups, fatal and non-fatal MACE, were used as endpoints. Correlation analyses were performed by Spearman correlation. The analyses were performed using IBM SPSS Statistics 22.

## Results

### Acute Phase

Characteristics of the cases and controls are presented according to acute phase serum MMP-9 quartiles (Table 1). CRP and smoking status differed statistically significantly between the quartiles in both groups. No other significant differences were observed. More detailed characteristics of the subjects have been presented previously [18]. The mean (SD) age of the cases and controls was 63.3 (9.2) and 63.0 (9.2) years and they included 21.3% and 22.1% women, respectively. Of the cases, 235 patients (68.5%) were diagnosed with AMI and 108 (31.5%) with UAP.

**Table 1:** Baseline characteristics of cases (acute phase) and controls in quartiles of serum MMP-9 concentrations

		Quartiles of serum MMP-9 concentrations^a^				
		1st	2nd	3rd	4th	
		Mean (SD)				*p* ^2^
Age (years)	Cases	63.7 (9.4)	63.2 (9.7)	62.7 (8.9)	63.7 (8.6)	NS
	Controls	62.5 (9.6)	62.8 (8.6)	63.7 (9.6)	63.1 (9.2)	NS
Cholesterol (mmol/l)	Cases	5.5 (1.1)	5.2 (1.6)	5.3 (1.3)	5.2 (1.0)	NS
	Controls	5.6 (1.0)	5.8 (1.1)	5.9 (1.0)	5.8 (1.1)	NS
CRP (mg/l)	Cases	12.6 (24.7)	14.6 (18.2)	23.1 (31.3)	53.3 (62.9)	*< 0.001*
	Controls	1.8 (1.7)	2.1 (2.2)	2.7 (2.9)	2.5 (2.5)	*0.035*
		*N* (%)				*p* ^3^
Sex (% men)	Cases	65 (76.5)	65 (75.6)	66 (76.7)	74 (86.0)	NS
	Controls	57 (70.4)	61 (74.4)	69 (84.1)	67 (82.7)	NS
Current smoker	Cases	12 (16.2)	7 (9.1)	14 (20.0)	22 (31.4)	*0.006*
	Controls	7 (9.5)	12 (15.0)	14 (17.5)	33 (41.3)	*< 0.001*
Diabetic	Cases	11 (13.1)	11 (13.3)	11 (13.4)	14 (16.5)	NS
	Controls	–	–	–	–	–
Lipid-lowering medication	Cases	15 (17.9)	22 (26.5)	23 (28.0)	17 (20.0)	NS
	Controls	–	–	–	–	–
MACE in follow-up	Cases	30 (35.3)	39 (45.3)	43 (50.0)	38 (44.2)	NS
	Controls	8 (9.9)	5 (6.1)	9 (11.1)	9 (11.1)	NS
Fatal	Cases	9 (10.6)	12 (14.6)	18 (20.9)	22 (25.6)	NS
	Controls	4 (4.9)	3 (3.7)	2 (2.5)	4 (4.9)	NS
Non-fatal	Cases	21 (24.7)	27 (31.4)	25 (29.1)	16 (18.6)	NS
	Controls	4 (4.9)	2 (2.4)	7 (8.5)	7 (8.6)	NS

Significant values are in italics

*NS* not significant

^a^Numbers of cases and controls in quartiles: 1st 85 and 81, 2nd 86 and 82, 3rd 86 and 82, and 4th 86 and 81

^b^ANOVA of log-transformed values

^c^Chi-square test

The serum MMP-9 and MMP-9/TIMP-1 molar ratio were significantly higher in ACS patients than in controls (*p* < 0.001) (Table 2; Supplement 1). The same difference was seen in both AMI (*p* < 0.001) and UAP (*p* < 0.001). Both MMP-9 (*p* < 0.001) and MMP-9/TIMP-1 (*p* = 0.023) were higher in AMI compared to UAP. The association of serum levels with ACS was investigated with multivariate logistic regression models, which are presented in Table 3. Both MMP-9 concentration and MMP-9/TIMP-1 were strongly associated with ACS and its subgroups.

**Table 2:** Median serum MMP-9 concentrations in the recovery phase relative to the acute phase

					MMP-9 (ng/ml)			
					Median (IQR)	*p* value		
				*N*		Compared to controls^a^	Compared to acute phase^b^	Compared to “no endpoint”^c^
Controls				326	150.2 (189.4)			
Cases	Acute phase	ACS		343	343.5 (298.7)	*< 0.001*	–	–
			UAP	108	302.4 (278.4)	*< 0.001*	–	–
			AMI	235	375.0 (330.4)	*< 0.001*	–	–
		MACE in the follow-up	No endpoint	193	322.2 (318.2)	*< 0.001*	–	–
			Non-fatal	89	327.6 (234.8)	*0.011*	–	*< 0.001*
			Fatal	61	419.3 (266.5)	*< 0.001*	–	*< 0.001*
	Recovery phase	ACS		157	174.6 (225.3)	NS	*< 0.001*	–
			UAP	56	178.2 (255.4)	NS	*0.013*	–
			AMI	101	172.9 (184.9)	NS	*< 0.001*	–
		MACE in the follow-up	No endpoint	94	182.4 (226.6)	NS	*< 0.001*	–
			Non-fatal	49	167.6 (214.0)	NS	*< 0.001*	NS
			Fatal	14	138.4 (295.9)	NS	*0.003*	NS

The statistically significant *p* values are in italics

*NS* not significant

^a^Mann-Whitney test

^b^Wilcoxon signed-rank test

**Table 3:** The association of serum MMP-9 and MMP-9/TIMP-1 quartiles with ACS at baseline

				OR (95% CI)				*p*
				1st	2nd	3rd	4th	
ACS	MMP-9		Model 1	1	1.92 (1.20–3.08)	5.62 (3.48–9.08)	10.37 (6.20–17.35)	< 0.001
			Model 2	1	1.64 (0.81–3.30)	4.84 (2.41–9.72)	5.81 (2.65–12.76)	< 0.001
	MMP-9/TIMP-1		Model 1	1	1.78 (1.12–2.82)	5.22 (3.25–8.37)	6.29 (3.87–10.22)	< 0.001
			Model 2	1	1.79 (0.87–3.67)	5.34 (2.61–10.91)	4.96 (2.37–10.38)	< 0.001
UAP		MMP-9	Model 1	1	1.81 (0.61–2.30)	3.72 (2.00–6.94)	4.49 (2.28–8.85)	< 0.001
			Model 2	1	0.89 (0.34–2.31)	3.09 (1.29–7.37)	3.81 (1.39–10.47)	0.004
		MMP-9/TIMP-1	Model 1	1	1.29 (0.66–2.52)	3.76 (1.98–7.13)	3.75 (1.93–7.26)	< 0.001
			Model 2	1	1.08 (0.40–2.89)	3.18 (1.28–7.89)	3.89 (1.55–9.74)	0.005
AMI		MMP-9	Model 1	1	2.70 (1.50–4.86)	7.65 (4.25–13.77)	16.82 (9.13–30.98)	< 0.001
			Model 2	1	3.01 (1.10–8.27)	9.05 (3.32–24.62)	10.97 (3.72–32.35)	< 0.001
		MMP-9/TIMP-1	Model 1	1	2.14 (1.23–3.70)	6.28 (3.62–10.87)	8.34 (4.76–14.61)	< 0.001
			Model 2	1	3.55 (1.26–10.00)	11.46 (4.09–32.06)	8.73 (3.03–25.10)	< 0.001

Model 1 stratified for age and sex (*N* = 654); model 2 stratified for age, sex, and adjusted for CRP, cholesterol concentration, and smoking (*N* = 504)

### Diagnostic Ability of Serum MMP-9 and MMP-9/TIMP-1

In ROC analyses, both MMP-9 and the MMP-9/TIMP-1 molar ratio distinguished ACS from the controls with an AUC (95%) of 0.742 (0.704–0.781, *p* < 0.001) and 0.702 (0.662–0.742, *p* < 0.001), respectively. Smoking decreased the diagnostic value of serum MMP-9 in ACS; in the ROC analysis, the sensitivity and specificity were higher in non-smokers than smokers with an AUC of 0.765 (0.722–0.808, *p* < 0.001) and 0.659 (0.560–0.758, *p* = 0.003), respectively. Serum MMP-9 had a significant correlation with CRP (*r* = 0.453, *p* < 0.001), CK-MB (0.278, *p* < 0.001), and troponin T (0.291, *p* < 0.001).

### Recovery Phase

Recovery phase samples were collected from ACS patients not earlier than 6 months after the acute event. Characteristics of patients in recovery phase and according to the difference of MMP-9 concentration between acute and recovery phase, i.e., ΔMMP-9, are presented in the supplementary material (Supplements 2 and 3). In the recovery phase relative to the acute phase, serum MMP-9 concentrations and MMP-9/TIMP-1 decreased 49 and 34% (*p* < 0.001), respectively (Table 2; Supplement 1). The recovery phase MMP-9 concentrations did not differ significantly from those of controls, but the MMP-9/TIMP-1 ratio remained significantly higher than the levels observed in controls (Table 2; Supplement 1).

### Follow-Up and Prognostics

The cases were followed up for 6 years, and MACE was registered. Cases experiencing MACE during follow-up had significant differences in CRP between the acute phase MMP-9 quartiles (CRP increasing in a dose-dependent manner). No other significant differences were observed in the characteristics between the quartiles (data not shown).

**Fig. 1:**
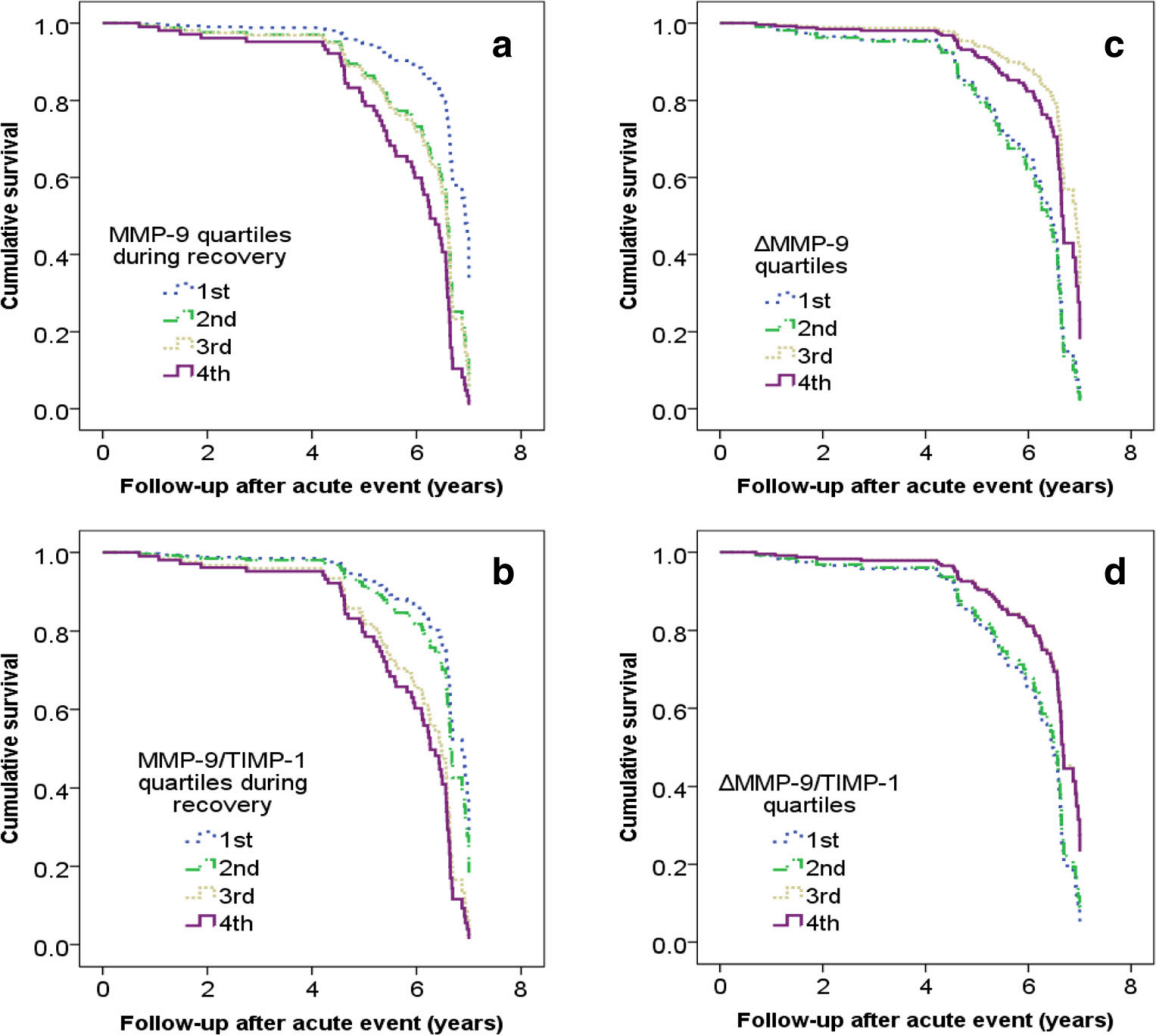
Cumulative survival according to the serum MMP-9, MMP-9/TIMP-1, ΔMMP-9, and ΔMMP-9/TIMP-1 quartiles in ACS patients, endpoint event being MACE. MACE includes both fatal and non-fatal endpoints. ΔMMP-9 and ΔMMP-9/TIMP-1 refer to difference between acute and recovery phase (i.e., acute—recovery phase values). The survival was investigated by Cox regression model adjusted for age and sex

We evaluated if serum MMP-9 and MMP-9/TIMP-1 molar ratio or their Δ values predict MACE. Elevated acute phase MMP-9 was a significant predictor of fatal events with a HR 2.88 (1.32–6.27, *p* = 0.025, Q4 vs. Q1), but not non-fatal ones. Elevated recovery phases MMP-9 and MMP-9/TIMP-1 were significant predictors of MACE with HRs 4.15 (1.87–9.23, *p* = 0.006) and 3.32 (1.48–7.42, *p* = 0.009) (Fig. 1), especially non-fatal events [6.05 (2.27–16.12), *p* = 0.004 and 6.89 (2.52–18.83), *p* = 0.001].

The highest ΔMMP-9 values presented protective predictive value of MACE with a HR 0.44 (0.20–0.97, *p* = 0.003) (Fig. 1) and especially non-fatal events [0.24 (0.09–0.59), *p* = 0.001]. Also, high ΔMMP-9/TIMP-1 was protective from non-fatal MACE with a HR 0.29 (0.11–0.73, *p* = 0.019).

### Activatable MMP-9

**Fig. 2:**
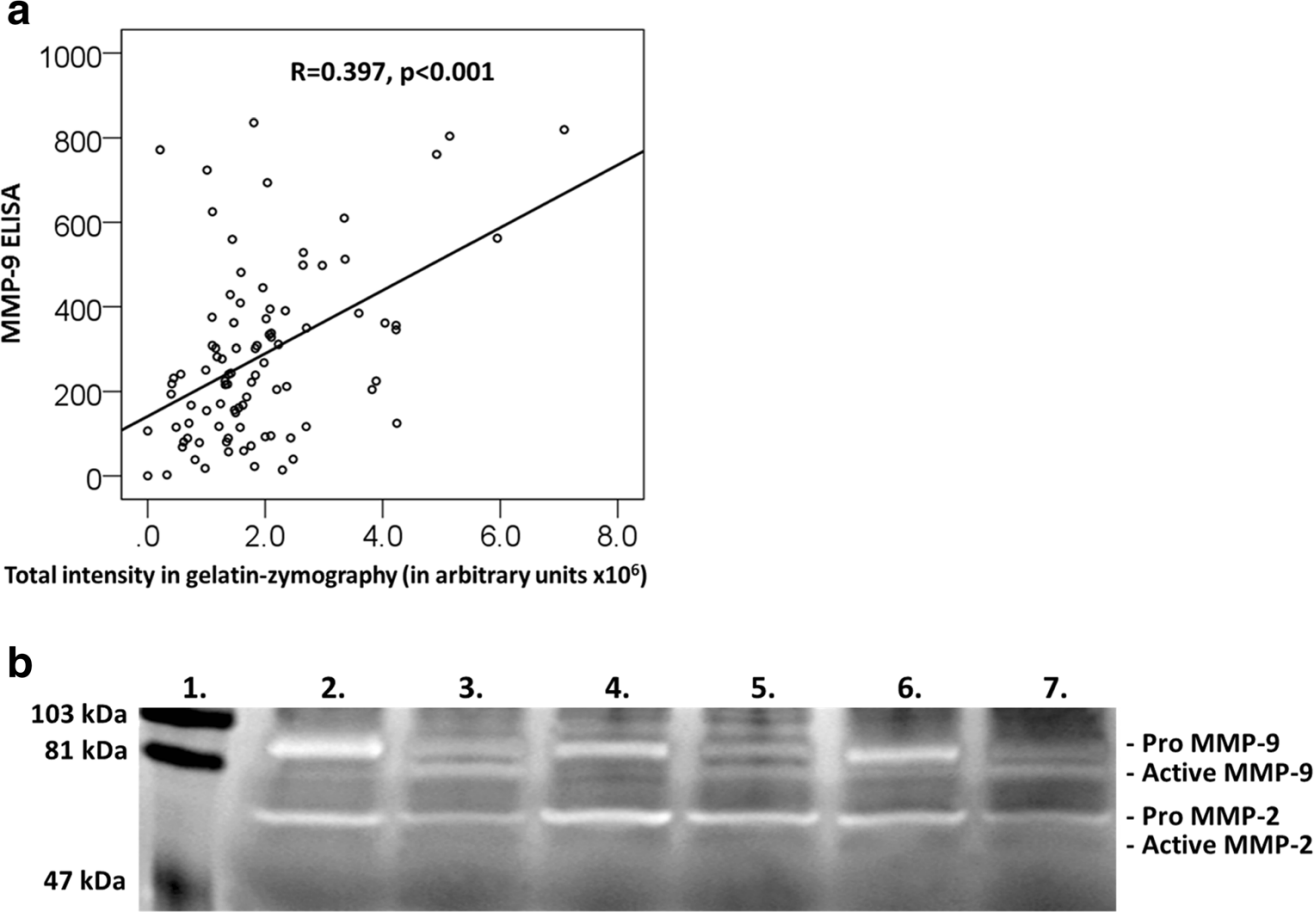
Gelatin zymography results. **a** Scatter plot of serum MMP-9 levels measured by ELISA and total MMP-9 intensities analyzed by gelatin zymography. The correlation coefficient and *p* value are shown. **b** Representative gelatin-zymography of ACS serum samples. Lane 1 is the molecular weight standard. Lanes 2, 4, and 6 are serum samples of patients with ACS without MMP-9 activating pretreatment. Lanes 3, 5, and 7 are the same serum samples, respectively, with 1 mM APMA pretreatment, which activates the pro-form of MMP-9. The gels are 10%, and the bands were visualized by Coomassie Brilliant Blue staining. Pro-MMP-2 bands are seen at 72 kDa. No proteolytically activated MMP-2 was observed (64 kDa)

Zymography was utilized to explore activatable MMP-9 in vitro. The serum MMP-9 concentrations obtained by ELISA correlated significantly with the total intensity units obtained by gelatin zymography (*r* = 0.397, *p* < 0.001) (Fig. 2a). In all samples, MMP-9 could be activated with APMA; the difference was significant for all comparisons (*p* ≤ 0.001 for all). Other gelatinolytic active MMP, MMP-2, was observed only in pro-form (72 kDa), but not in proteolytically activated form (64 kDa) (Fig. 2b). High molecular size (> 100 kDa) gelatinolytic species were also detected.

**Fig. 3:**
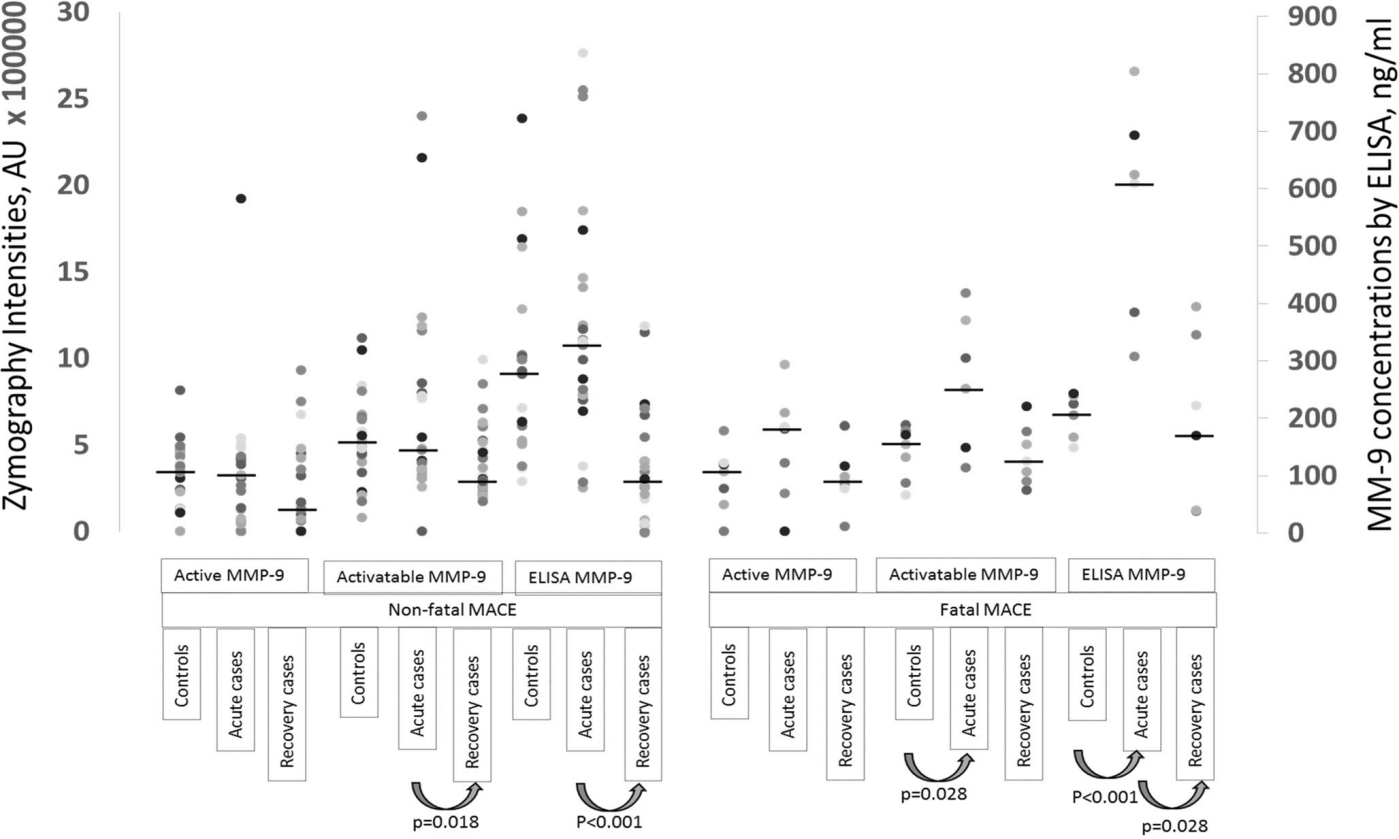
Active and APMA-activatable MMP-9 analyzed by gelatin zymography presented as arbitrary units of intensities (Y1-axis) and corresponding MMP-9 concentrations measured by ELISA (Y2-axis) in the subjects selected for zymography. All measurement points are presented as dots, and group medians are presented with a line. Statistically significant differences are presented below the plot

Medians of active and APMA-activatable MMP-9 were compared between cases and controls. When cases were analyzed as one group (endpoint being MACE), there were no statistically significant differences in the active and activatable MMP-9 between the cases and controls in acute phase. However, both molecular forms of MMP-9 decreased in the recovery phase (*p* = 0.179 for active and *p* = 0.003 for activatable) below the levels observed in controls. Additionally, values were calculated separately in cases with different endpoints both in the acute and recovery phase (Fig. 3). In cases with different endpoints, active and activatable MMP-9 decreased from the acute phase to the recovery phase, but the difference was significant only for activatable MMP-9 in cases suffering a non-fatal MACE (*p* = 0.018) (Fig. 3). Patients with fatal MACE during follow-up had significantly higher activatable MMP-9 zymography intensities (*p* = 0.028) and MMP-9 ELISA concentrations (*p* < 0.001) in acute phase than controls (Fig. 3). In the zymography subpopulation, MMP-9 concentrations obtained by ELISA differed statistically significantly between acute and recovery phase in cases with non-fatal (*p* < 0.001) and fatal MACE (*p* = 0.028) (Fig. 3).

Proteolytically activated MMP-2 lanes (64 kDa) were not observed in our zymography analysis, only MMP-9 was proteolytically activated (Fig. 2b). Medians of pro-MMP-2 (72 kDa) were significantly higher in cases with a fatal MACE when compared to controls both without (*p* = 0.013) and with (0.022) APMA-activation and when compared to non-fatal MACE without (*p* = 0.016) and with (*p* = 0.025) APMA-activation (Supplement 4). The corresponding values did not differ significantly between non-fatal MACE and controls.

## Discussion

Serum MMP-9 concentrations were elevated in the acute phase of ACS and they generally decreased during the recovery. The high levels during the acute phase predicted fatal MACE. Patients with high MMP-9 concentrations or MMP-9/TIMP-1 ratio that persisted during the course of recovery were at increased risk for MACE, especially for non-fatal events. As novel findings, the largest decrease of MMP-9 concentrations between acute and recovery phase (Δ values) was protective against MACE, especially non-fatal events. When examining the molecular forms of MMP-9, the cases with a fatal outcome had during the acute phase the highest activatable MMP-9 values.

The association of MMP-9 with different cardiac outcomes has been previously reported; plasma MMP-9 is associated with CVD mortality in patients with CAD at baseline [16, 23]. Our results are in agreement with these findings; acute phase MMP-9 concentration predicted especially CVD death in the 6-year follow-up. Earlier studies have also suggested that elevated MMP-9 is associated with higher risk of death due to any cause [24] and with CVD risk factors and total cardiovascular risk in subjects without symptoms of CAD [25]. In our study, the recovery phase MMP-9 concentrations and MMP-9/TIMP-1 ratio were better predictors of future outcome than the levels measured at the baseline. Importantly, the more these levels decreased after the acute phase, the better was the predictable outcome, especially regarding non-fatal events. Similar approach was used in a 30-day follow-up of stroke patients [26], where the serum MMP-9 concentration in the follow-up correlated positively with initial stroke severity and outcome. In this study during the short follow-up, the MMP-9 concentrations did not reach the levels observed in the controls and the Δ values were not as valuable as in our study.

In our study, increased serum MMP-9 concentrations were associated with both AMI and UAP, which confirms previous findings [14]. In patients with STEMI, ACS, and non-STEMI [27], the MMP-9 concentrations elevate earlier than the classical myocardial damage marker, high-sensitivity troponin T, presumably reflecting plaque rupture. MMP-9 concentrations were highest after admission to hospital in patients with non-STEMI but decreased rapidly during the following 48 h [28]. MMP-9 had a significant correlation with myocardium-specific markers CK-MB and troponin T, suggesting that ischemic myocardial tissue is a source of systemic MMP-9 after an ACS event. Myocardial damage can induce enhanced MMP expression and activation [29]. In our study, elevated MMP-9 levels were found also during the recovery phase, which suggests that serum MMP-9 may derive also from other source than plaque rupture. Cytokines and other pro-inflammatory mediators increase the synthesis and secretion of MMP-9 from inflammatory cells [3]. CRP increases MMP-9 expression in smooth muscle cells in a dose-dependent manner and correlates with MMP-9 levels in ACS patients [30]. This correlation was also observed in the present study. Serum MMP-9 may reflect a systemic inflammatory state [1], but genetic variation may also contribute to the MMP-9 levels and activity [31] and thereby to the risk of coronary artery disease [32].

Serum MMP-9 and MMP-8 share common pathways in the pathophysiology of ACS. We have published earlier the results on serum MMP-8 and MMP-8/TIMP-1 in the present population, which offers a chance to compare these two biomarkers. The MMP-9 concentrations and MMP-9/TIMP-1 molar ratio differed between cases and controls similar to MMP-8 and MMP-8/TIMP-1 and had strong associations with ACS [18]. Both MMP-9 and MMP-8 decreased in a similar manner in the course of recovery [18]. However, the prognostic value of MMP-9 and MMP-8 differed from each other markedly: the acute phase MMP-9 was a predictor of cardiovascular death and the recovery MMP-9 of hospitalization for an ACS event, while MMP-8 had no prognostic value for these end points in the present population [18].

Upregulated TIMP-1 may suppress MMP-8 and MMP-9 [33], thus providing an important regulatory step in physiological circumstances. In our previous study, acute and recovery phase TIMP-1 concentrations were associated with cardiovascular death with hazard ratios of 4.31 (*p* < 0.001) and 4.69 (*p* = 0.037), respectively [18]. In the present study, MMP-9/TIMP-1 molar ratio determined in the recovery phase predicted MACE. Therefore, the balance between MMPs and TIMPs may be crucial for the destabilization of atherosclerotic plaques in acute phase [18], since MMP inhibitors have been shown to reduce myocardial infarct size [34]. Synthetic MMP inhibitors, such as ilomastat, could be cardioprotective, especially in preventing reperfusion injury [35]. However, there are challenges in optimizing the pharmacological targeting, since MMP-9 is required also in the healing processes [36].

Investigation of MMP-9 activation and activation potential may offer new insights in MMP-9 diagnostics and prognostics, even if challenges remain. Gelatin zymography is a widely used technique for detecting active MMP-9 based on molecular weight [37]. MMP-9 is expressed as a 92 kDa pro-form, whose molecular weight upon activation usually decreases by 10 kDa [22]. High molecular size (> 100 kDa) gelatinolytic species represent MMP-9 clustered together with the neutrophil gelatinase associated lipocalin (NGAL) [38] and MMP-9 linked with TIMP-1 [39]. Patients with cardiovascular death had significantly increased acute phase activatable MMP-9 values when compared to controls. Interestingly, the activatable MMP-9 decreased significantly between the acute and recovery phase in cases suffering a non-fatal MACE. Thus, the proportion of molecular forms and “activation potential” may be crucial for prognostics. MMP-9 can be activated by several proteolytic and non-proteolytic ways, and the alternative MMP-9 activation does not necessarily involve the molecular weight change [7, 40]. This may at least in part explain why the amounts of active MMP-9 analyzed by gelatin zymography did not differ significantly between cases and controls in the acute phase. On the other hand, in vitro conversion was observed in all samples when serum MMP-9 was processed chemically with APMA. In gelatin zymography, the functional activity of MMP-9 under physiological conditions cannot be thoroughly addressed, as it is not clear whether SDS disrupts the normal physiological interactions between MMP-9 and TIMP-1 [41].

MMP-2 is other gelatinolytic MMP, which is related to inflammation in myocardium [42]. MMP-2 is usually activated with proteolysis and molecular weight drops from 72 to 64 kDa [43]. In our small zymography subpopulation, we noticed differences in the full size MMP-2 between cases and controls, but proteolytically activated MMP-2 band was not present. Oxidative stress can also activate MMP-2 and MMP-9, and in a pilot study, serum nitrotyrosine, a marker describing oxidative stress, correlated with active MMP-9, but not with active MMP-2 [44]. The present study confirms and further extends earlier findings regarding the importance of MMP-9 in CAD.

The diagnostic efficacy and accuracy of serum MMP-9 needs to be further explored. In our study, diabetes did not associate with serum MMP-9 levels. While this observation differs from earlier findings [45], the small number of diabetic subjects may have affected these results. Also, other systemic conditions such as obesity [46] and metabolic syndrome [47] elevate serum MMP-9 concentrations. Smoking is a strong confounding factor in MMP-9 diagnostics; similar trend using serum MMP-8 in ACS-diagnostics was previously documented [19].

There are several limiting factors in our study. We cannot estimate the proportion of MMP-9 excreted from ischemic myocardium or ruptured atherosclerotic plaques from serum analysis. In addition, we do not have information on the infarct size; thus, the models cannot be adjusted by it. Nevertheless, the hazard ratio for a non-fatal MACE measured from recovery phase samples strongly supports the hypothesis that the elevation of MMP-9 in these patients was mainly due to the cardiac events. In zymography analysis, we cannot calculate the total physiological MMP-9 activity. The serum samples were collected in 1999–2002, when the use of statins was not common, and we have information only on lipid-lowering medication. Statins may affect MMP-9 concentrations [48], albeit in meta-analyses statins had no significant effect on plasma MMP-9 [49]. Plasma TIMP-1 concentrations decreased after use of statins [49]. We could not adjust the models with BMI and blood pressure, as we did not have this information on our control population. However, according to our inclusion criteria, the controls were not using anti-hypertensive medication.

Novel biomarkers are needed to identify CVD risk patients, early diagnostics of atherosclerotic plaque rupture or ischemia, and CVD prognostics. Serum MMP-9 determination is sensitive and specific, thus being a good candidate as a biomarker in clinical practice. MMP-9 plays a role both in atherosclerotic plaque rupture and tissue destruction after a cardiac event. The balance between MMP-9 and TIMP-1 may be crucial for disease progression. The difference of MMP-9 and MMP-9/TIMP-1 between acute and recovery phase provide novel prognostic information of secondary cardiac events. Additionally, the analysis of MMP-9 activation potential may offer new insights into cardiac diagnostics and prognostics.

### Clinical Relevance

MMP-9 can be utilized as an early stage biomarker, because its elevation reflects atherosclerotic plaque rupture and myocardial tissue destruction. Furthermore, MMP-9 has prognostic value, which is important in secondary prevention and planning of personalized treatment.

## Electronic Supplementary Material

ESM 1 (DOCX 65 kb)
