# Supplementary material for: Serum MMP-9 Diagnostics, Prognostics, and Activation in Acute Coronary Syndrome and Its Recurrence
Source: J Cardiovasc Transl Res. 2018 Jan 18;11(3):210–20. doi: 10.1007/s12265-018-9789-x (PMC5974001; doi:10.1007/s12265-018-9789-x)
Supplement: Supplementary file 1 — (DOCX 65 kb) [file 12265_2018_9789_MOESM1_ESM.docx]

**Electronic Supplementary Material**

**Serum MMP-9 diagnostics, prognostics, and activation in acute coronary syndrome and its recurrence**

**Laura Lahdentausta^1^*,** Jaakko Leskelä^1^, Alina Winkelmann^2^, Taina Tervahartiala^1^, Timo Sorsa^1,3^, Erkki Pesonen^4,5^ and Pirkko J Pussinen^1^

1 Department of Oral and Maxillofacial Diseases, University of Helsinki and Helsinki University Hospital, Finland

2 Department of Periodontology, Operative and Preventive Dentistry, University Hospital Bonn, Bonn, Germany

3 Division of Periodontology, Department of Dental Medicine, Karolinska Institutet, Huddinge, Sweden

4 Skåne University, Sweden

5 Department of Paediatrics, Division of Paediatric Cardiology, Skåne University Hospital, Lund, Sweden

**Supplement 1. Median MMP-9/TIMP-1 ratios in the recovery phase relative to the acute phase**

|  |  |  |  | | **MMP-9/TIMP-1** |  | | |
| --- | --- | --- | --- | --- | --- | --- | --- | --- |
|  |  |  |  | | **Median (IQR)** | **p value** | | |
|  |  |  |  | N |  | Compared to controls^1^ | Compared to acute phase^2^ | Compared to “no endpoint”^1^ |
| **Controls** |  |  |  | 326 | 0.40 (0.54) |  |  |  |
| **Cases** | Acute phase | ACS |  | 343 | 0.85 (0.87) | **<0.001** | - | - |
|  |  |  | UAP | 108 | 0.79 (0.78) | **<0.001** | - | - |
|  |  |  | AMI | 235 | 0.90 (0.94) | **<0.001** | - | - |
|  |  | MACE in the follow-up | No endpoint | 193 | 0.85 (1.01) | **<0.001** | - | - |
|  |  |  | Non-fatal | 89 | 0.88 (0.70) | **0.026** | - | **<0.001** |
|  |  |  | Fatal | 61 | 0.84 (0.71) | **0.001** | - | **0.001** |
|  | Recovery phase | ACS |  | 157 | 0.56 (0.84) | **<0.001** | **<0.001** | - |
|  |  |  | UAP | 56 | 0.63 (0.89) | **<0.001** | NS | - |
|  |  |  | AMI | 101 | 0.55 (0.79) | **0.006** | **<0.001** | - |
|  |  | MACE in the follow-up | No endpoint | 94 | 0.57 (0.83) | **<0.001** | **0.015** | - |
|  |  |  | Non-fatal | 49 | 0.65 (0.73) | NS | NS | NS |
|  |  |  | Fatal | 14 | 0.38 (0.88) | NS | NS | NS |

^1^ Mann-Whitney test; ^2^ Wilcoxon signed-rank test, NS= not significant, the statistically significant p-values are bolded

**Supplement 2. Characteristics of cases in recovery phase**

|  | Quartiles of recovery phase MMP-9 ^1^ | | | |  |
| --- | --- | --- | --- | --- | --- |
|  | **1^st^** | **2^nd^** | **3^rd^** | **4^th^** |  |
|  | **Mean (SD)** | | | | **p^2^** |
| Age (years) | 68.1 (8.3) | 61.0 (7.6) | 63.1 (9.0) | 63.7 (8.2) | **0.005** |
| Cholesterol (mmol/l) | 5.3 (1.0) | 5.0 (1.0) | 5.2 (1.0) | 5.4 (2.0) | NS |
| CRP (mg/l) | 10.3 (12.4) | 24.2 (48.0) | 31.9 (42.7) | 17.3 (35.0) | **0.012** |
|  | **N (%)** | | | | **p^3^** |
| Sex (% men) | 32 (84.2) | 33 (82.5) | 34 (87.2) | 30 (75.0) | NS |
| Current smoker | 2 (5.6) | 3 (7.7) | 7 (18.9) | 9 (22.5) | NS |
| Diabetic | 6 (16.2) | 4 (10.0) | 1 (2.6) | 5 (12.8) | NS |
| Lipid lowering-medication | 6 (16.2) | 9 (22.5) | 10 (26.3) | 9 (23.1) | NS |
| MACE in follow-up | 17 (44.7) | 17 (42.5) | 13 (33.3) | 16 (40.0) | NS |
| Fatal | 5 (13.2) | 3 (7.5) | 2 (5.3) | 4 (10.3) | NS |
| Non-fatal | 12 (31.6) | 14 (35.0) | 11 (28.2) | 12 (30.0) | NS |

^1^Number of cases in quartiles: 1^st^ 38, 2^nd^ 40, 3^rd^ 39, 4^th^ 40; ^2^ANOVA of log-transformed values; ^3^Chi-square test; NS= not significant, significant values are bolded

**Supplement 3. Characteristics of cases according to quartiles of** Δ **MMP-9 (acute phase - recovery phase)**

|  | Quartiles of Δ MMP-9 ^1^ | | | |  |
| --- | --- | --- | --- | --- | --- |
|  | **1^st^** | **2^nd^** | **3^rd^** | **4^th^** |  |
|  | **Mean (SD)** | | | | **p^2^** |
| Age (years) | 64.2 (7.5) | 63.2 (9.8) | 65.3 (8.1) | 63.0 (9.0) | NS |
| Cholesterol (mmol/l) | 5.4 (1.9) | 5.3 (1.2) | 5.0 (1.1) | 5.3 (0.9) | NS |
| CRP (mg/l) | 10.0 (13.0) | 12.2 (14.1) | 19.7 (25.8) | 40.7 (64.0) | **0.016** |
|  | **N (%)** | | | | **p^3^** |
| Sex (% men) | 29 (74.4) | 31 (79.5) | 33 (82.5) | 36 (92.3) | NS |
| Current smoker | 7 (18.4) | 5 (12.8) | 2 (5.3) | 7 (18.9) | NS |
| Diabetic | 4 (10.5) | 3 (7.9) | 4 (10.3) | 5 (12.8) | NS |
| Lipid lowering-medication | 7 (18.4) | 11 (28.9) | 9 (23.1) | 7 (17.9) | NS |
| MACE in follow-up | 15 (38.5) | 19 (48.7) | 12 (30.0) | 17 (43.6) | NS |
| Fatal | 2 (5.3) | 4 (10.5) | 2 (5.0) | 6 (15.4) | NS |
| Non-fatal | 13 (33.3) | 15 (38.5) | 10 (25.0) | 11 (28.2) | NS |

^1^Number of cases in quartiles: 1^st^ 39, 2^nd^ 39, 3^rd^ 40, 4^th^ 39; ^2^ ANOVA of log-transformed values; ^3^ Chi-square test; NS = not significant, significant values are bolded

**Supplement 4. Pro-MMP-2 (72 kDa) medians (IQR) of zymography intensities (UI)**

|  | Acute, non-APMA | Acute, APMA |
| --- | --- | --- |
| Controls, no endpoint (N=28) | 467500 (564000) | 310000 (468500) |
| Cases with fatal MACE (N=7) | 1200000 (1744000), *p=0.013 | 693000 (580000), *p=0.022 |
| Cases with non-fatal MACE (N=23) | 423000 (889400), **p=0.016 | 304000 (444000), **p=0.025 |

*p-value compared to control group tested by non-parametric Mann-Whitney test

**p-value compared to fatal MACE group tested by non-parametric Mann-Whitney test
